# Inequities in access to depression treatment: results of the Brazilian National Health Survey – PNS

**DOI:** 10.1186/s12939-016-0446-1

**Published:** 2016-11-17

**Authors:** Claudia Souza Lopes, Natália Hellwig, Gulnar de Azevedo e Silva, Paulo Rossi Menezes

**Affiliations:** 1 Institute of Social Medicine, State University of Rio de Janeiro, Rio de Janeiro, Brazil; 2 Department of Preventive Medicine, University of São Paulo, São Paulo, Brazil

**Keywords:** Depression, Health surveys, PHQ-9, Mental health, Mental health service access

## Abstract

**Background:**

Despite depression being one of the most prevalent mental disorders in the world, access to treatment is still insufficient, especially in low- and middle-income countries. The aim of this study is to investigate differences in access to treatment for depression according to socio-demographic characteristics, geographical area and multi-morbidity in a nationally representative sample of individuals with depression.

**Methods:**

This study analyses data from the National Health Survey (*Pesquisa Nacional de Saúde* – PNS), a Brazilian household-based nationwide survey, which comprises 60,202 adults (aged 18 years or older). Depression was evaluated through the *Patient Health Questionnaire-9* (PHQ-9). Prevalence Ratios and corresponding 95 % confidence intervals (95%CI) were calculated using Poisson regression.

**Results:**

The general prevalence of depression was 7.9 % (95 % CI 7.5 to 8.3). Among those with depression, 78.8 % did not receive any treatment, and 14.1 % received only pharmacotherapy. Multivariable analyses showed that being female, white, aged between 30 and 69 years, living in regions other than the North, having higher education and having multi-morbidities were independently associated with higher likelihood of access to any treatment.

**Conclusions:**

Most Brazilians with clinically relevant depressive symptoms are not receiving any treatment. Access to care is unequal, with the poor and those living in low resource areas having higher difficulties to access mental health care. Understanding these disparities is important for the provision of effective interventions aimed at reducing the prevalence of depression and inequities in access to mental health care.

## Background

Depression is one of the most important mental disorders, both due to its high prevalence worldwide and also because of the commonly chronic course of its presentation (leading to a high lifetime prevalence), with a significant burden for individuals’ lives and public health systems [1, 2]. Data from the Global Burden of Disease Study – 2010 showed that depression is the leading contributor, accounting for 2.5 % of Disability Adjusted Life Years (DALYs), and the second leading cause of disability, accounting for 8.2 % of Years Lived with Disability (YLDs). Depression was also considered the main cause of 16 million suicide DALYs and almost 4 million ischaemic heart disease DALYs [3].

Effective treatment for depression includes antidepressant medications and psychotherapies, either alone or in combination [4]. However, a large proportion of those with depression do not receive any type of care. It is estimated that 35 to 50 % of individuals with severe depressive symptoms in high-income countries do not receive any treatment [5, 6]. Moreover, among those receiving care for depression, only about 20 % get effective treatment. In low- and middle-income countries the situation is even worse, with only 15 to 25 % of those with severe depressive symptoms receiving any treatment [5]. Lack of specialized human resources and adequate budgets for mental health care account for a great deal of this huge deficiency [6]. This treatment gap is marked by social and geographical disparities, those who most need care, such as the poor and those living in where regions with limited mental health resources having greater difficulty receiving adequate care for depression.

In Brazil, a 2013 population-based survey with a nationally representative sample, the National Health Survey (PNS), showed that the prevalence of major depression is higher among women, those living in urban areas, those with lower educational levels, and those with chronic conditions, such as hypertension and diabetes [7]. The data also showed that the lowest prevalence was observed in the Northern region, whereas the highest prevalence was found in the Southern region.

In present study, we used the PNS data to examine the extent of the treatment gap for depression in Brazil and associated inequities. We aimed to: 1) estimate the proportion of Brazilians with clinically relevant depressive symptoms who have access to treatment; and 2) evaluate differences in access to treatment according to sociodemographic characteristics, geographical area and presence of multi-morbidity among those presenting clinically relevant depressive symptoms.

## Methods

### Study design and sample

This study analyzed data from the National Health Survey (PNS), a household-based nationwide survey conducted by the Ministry of Health, in partnership with the Brazilian Institute of Geography and Statistics (IBGE), in 2013. The scope of the survey was to establish the health status and lifestyles of the population - as well as to examine aspects of access to and use of preventive and therapeutic services, continuity of care and health care financing.

The survey sample was designed to allow for the estimation of indicators for Brazil and at different geographic levels, namely major regions, states, capitals, and metropolitan and rural areas. The sampling design was by clusters in three stages: In the first stage, census tracts or set of sectors were selected to from the primary sampling units (PSUs). In the second stage, households were randomly selected within each PSU. In the third stage, an adult resident (18 years old or older) was selected with equal probability among all adult residents in the household. Weighting factors were calculated for each of the three sampling units, considering the probabilities of selection and the non-response rate. For the selected resident, the weighting factor was calculated considering the weight for the corresponding household, the probability of selection of the resident, the adjustment of non-response for sex, and calibration for the total population by sex and age groups estimated with the weight of all residents. More information about the design and methodology of the study, including the sampling process, can be obtained in previous publications [8, 9].

After forecasting a rate of 23 % non-response, the estimated sample size needed was 81,357 households. Losses were considered when households were closed or uninhabited; residents refused to answer the interview; or residents were not found after three or more attempts, even with scheduling visits. In the final survey, a total of 69,954 occupied households were visited and 60,202 individuals were interviewed, resulting in a response rate of 86.1 %.

The PNS project was approved by the National Commission of Ethics in Research (CONEP) in June 2013, Regulation No. 328.159, and all participants signed an informed consent agreement.

### Measures

Data were collected by research assistants, supervisors and coordinators of the IBGE, through a structured questionnaire using a Personal Digital Assistant (PDA). The questionnaire addressed a variety of health issues, lifestyle, socio-demographic and economic characteristics, among others. For the present study, we analyzed data focused on depressive symptoms, physical morbidity, use of health services and socio-demographic characteristics (gender, age, marital status, educational level and skin color).

### Measurement of depression

For the evaluation of depression, we used the Brazilian version of the *Patient Health Questionnaire* (PHQ-9) [10], which is the depression module of the *Primary Care Evaluation of Mental Disorders* (PRIME-MD) [11]. Each item of the PHQ-9 covers one symptom of depression according to criteria for the diagnosis of depression established by the *Diagnostic and Statistical Manual of Mental Disorders, Fourth Edition* (DSM-IV). For each item, possible answers and respective scores are: (0) ‘not at all’, (1) ‘less than half of the days’, (2) ‘more than half of the days’, and (3) ‘almost every day’. Total PHQ-9 scores of 5–9, 10–14, 15–19 and 20 or more represent mild, moderate, moderately severe, and severe depression, respectively [12].

A population-based validation study, conducted in a medium-sized Brazilian city, demonstrated that the PHQ-9 exhibited good validity in diagnosing major depression at the cut-offs of ≥ 9 and ≥ 10. The same study found that the PHQ-9 had a sensitivity of 72.5 % (95 % confidence interval (CI): 61.5 to 89.2) and a specificity of 88.9 % (95 % CI: 83.0 to 89.9) in identify major depression using the cut-off point ≥ 10 [10].

In the present study, depression was defined by a PHQ-9 score of 10 or higher, which is considered as the presence of clinically relevant depressive symptoms that can benefit from treatment (pharmacotherapy and/or psychotherapy) [12].

### Access to treatment for depression

For the assessment of access to treatment by those presenting depression, we used a question that asked whether the individual was currently seeing a doctor or health service regularly for depression. For those who answered “yes”, another question was used to classify the type of treatment received (psychotherapy, medication, or both). For those who answered that they were receiving medication, further questions assessed if these were acquired at a public health service, through some type of health plan or insurance, or whether it was paid for by the individual out of pocket.

### Predictors of treatment

Socio-demographic characteristics investigated were: gender (male and female); age group (18–29; 30–39; 40–49; 50–59; 60–69; 70 years or over); race/skin color; level of education (uneducated or incomplete primary school; complete primary school or incomplete high school, complete high school or incomplete college/university, complete college/university); and marital status (married or living with a partner vs. single). Geographical areas were defined as macro-region of residence (North, Northeast, Center-west, Southeast, and South), and living in urban vs. rural areas. Multi-morbidity was evaluated through self-reported physician diagnosis of the following conditions: arterial hypertension, diabetes, cardiac diseases, stroke, asthma, arthritis/rheumatism, back pain, chronic obstructive pulmonary disease, renal failure, cancer, musculoskeletal disorder related to working, other mental illnesses and other chronic diseases. Individuals were classified as having none, one, two or three or more of these conditions.

### Statistical analysis

Initially, a descriptive analysis was carried out and the resulting frequencies were adjusted according to the sample design and expanded for the Brazilian population. Prevalence of depression was described according to socio-demographic characteristics, geographical area and multi-morbidity. Among those with depression, the type of treatment received and the acquisition of medicines were presented stratified by geographic region of residence. For the evaluation of predictors of treatment, a multivariate analysis was carried out using Poisson regression, and prevalence ratios of access to treatment with their 95 % confidence intervals for the association between socio-demographic characteristics, geographical area of residence and multi-morbidity and access to treatment for depression were estimated. Crude and adjusted analyses were performed. Variables that presented a p-value of 0.20 in each level were maintained in the adjusted analysis. Analyses were performed using the Stata 14.0 statistical package. The survey *svyset* command was used to account for the effects of the complex design and included final sampling weights (Stata Corp. College Station, United States).

## Results

Table 1 shows the characteristics of the study population, the weighted projection for the Brazilian adult population and the prevalence of depression according to individual characteristics. The sample was composed of 60,202 individuals, of whom 52.9 % were female, 47.5 % declared that their skin color was white, more than 86 % lived in urban areas, 47.1 % were between 18 and 39 years old, 43.8 % lived in the Southeast region, 61.2 % lived with a partner, 38.3 % were uneducated or had incomplete primary education, and 47.9 % of subjects had no morbidity. The prevalence of depression was 7.9 % (95 % CI 7.5 to 8.2), corresponding to 11,553,035 (95 % CI 10,970,345 to 12,135,726) individuals.

**Table 1:** Sociodemographic characteristics, multimorbidity and prevalence of depression (PHQ-9 ≥ 10) for the Brazilian adult population. PNS/Brazil, 2013. (*n* = 60,202; weighted *n* = 146,308,458)

Variables				Depression	
	n	Weighted n^a^	%	Weighted n^a^	%
Gender					
Male	25,920	68,916,470	47.1	3,255,511	4.7
Female	34,282	77,391,988	52.9	8,297,524	10.7
Age					
18 to 29	14,321	38,157,850	26.1	2,174,491	5.7
30 to 39	14,269	31,643,091	21.6	2,314,648	7.3
40 to 49	11,405	26,423,124	18.0	2,305,212	8.7
50 to 59	9,030	23,676,562	16.2	2,294,739	9.7
60 to 69	6,238	14,894,253	10.2	1,277,645	8.6
70 or more	4,939	11,513,578	7.9	1,186,299	10.3
Education					
None or incomplete primary school	24,083	57,041,784	39.0	5,841,390	10.2
Complete primary school or incomplete high school	9,215	22,761,619	15.5	1,776,611	7.8
Complete high school or incomplete college/university	19,149	48,109,933	32.9	2,871,793	6.0
Complete college/university	7,603	18,395,122	12.6	1,075,825	5.8
Race/skin color					
Black	5,631	13,454,163	9.2	1,150,680	8.6
White	24,106	69,441,261	47.5	5,198,101	7.5
Asian	533	1,371,822	0.9	105,306	7.7
Brown	29,512	61,418,883	42.0	5,048,783	8.2
Indigenous	417	619,019	0.4	50,165	8.1
Marital status					
Married or living with a partner	34,522	89,537,328	61.2	6,899,008	7.7
Single or no partner	25,680	56,771,130	38.8	4,654,027	8.2
Macro-region					
North	12,536	10,885,968	7.4	659,268	6.1
Northeast	18,305	38,947,575	26.6	3,121,942	8.0
Center-west	7,519	10,775,569	7.4	885,227	8.2
Southeast	14,294	64,074,682	43.8	4,913,627	7.7
South	7,548	21,624,664	14.8	1,972,971	9.1
Geographical area					
Urban	49,245	126,132,422	86.2	10,260,840	8.1
Rural	10,957	20,176,036	13.8	1,292,195	6.4
Multimorbidity					
None	33,768	80,093,878	54.8	3,003,770	3.8
One	15,378	37,632,350	25.7	3,210,081	8.5
Two	6,749	17,328,645	11.8	2,384,125	13.8
Three or more	4,307	11,253,585	7.7	2,955,058	26.3

^a^Estimated values for Brazilian adult population

**Fig. 1:**
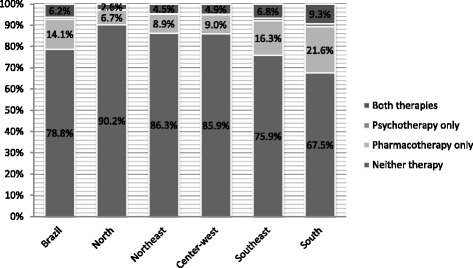
Receipt of treatment between depressed individuals. Brazil and regions. PNS/Brazil, 2013. (*n* = 5.501)

**Fig. 2:**
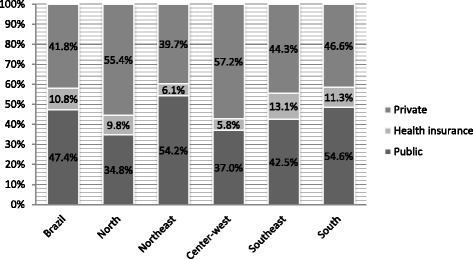
Type of acquisition between depressed individuals who were receiving medication. Brazil and regions. PNS/Brazil, 2013. (*n* = 619)

Figure 1 shows the type of treatment received among individuals with depression. The proportion of individuals not receiving any treatment was almost 80 % in Brazil. The Northern region had the highest proportion of untreated individuals (over 90 %), and the Southern region, the lowest proportion (67.5 %). The most common type of treatment was pharmacotherapy only, in all regions (around 14 % in Brazil); the Southern region had the highest proportion of individuals who received pharmacotherapy alone (21.6 %) or in addition to psychotherapy (9.3 %). Regarding the acquisition of medicines among individuals who had depression and received pharmacotherapy, 47.4 % acquired it through the public system, 41.8 % had to buy it, and just over 10 % acquired their medicines through a private health plan/insurance (Fig. 2). The highest proportions of users acquiring medications through the public system were observed in the Southern and Northeastern regions.

Table 2 includes crude and adjusted prevalence ratios of associations between socio-demographic characteristics, geographical areas and multi-morbidity and access to treatment among participants with depression. In the bivariate analysis, only marital status was not significantly associated with receiving any type of depression treatment. Multivariable Poisson regression showed that being female, white, between 30 to 69 years old, living in a macro-region other than the North, having a college/university degree and having multi-morbidities were all independently associated with higher likelihood of having access to treatment for depression.

**Table 2:** Crude and adjusted analyses of the associations between sociodemographic characteristics and multimorbidity with access to treatment among individuals with depression. PNS/Brazil, 2013. (*n* = 5.051)

Variables	No treatment (%)	Crude PR^a^ (95 % CI)	Adjusted PR^b^ (95 % CI)
Gender			
Male	15.2	1.00	1.00
Female	23.5	1.63 (1.37-1.93)	1.42 (1.14-1.77)
Race/skin color			
Black	16.3	1.00	1.00
White	25.3	1.69 (1.29-2.23)	1.66 (1.22-2.26)
Asian	23.2	0.94 (0.43-2.09)	1.48 (0.72-3.02)
Brown	18.0	1.26 (0.96-1.65)	1.30 (0.94-1.79)
Indigenous	25.7	1.94 (0.79-4.75)	2.49 (1.60-3.86)
Geographical area			
Urban	21.7	1.54 (1.26-1.88)	1.03 (0.85-1.25)
Rural	17.3	1.00	1.00
Age			
18 to 29	12.8	1.00	1.00
30 to 39	20.7	1.93 (1.50-2.50)	1.69 (1.30-2.20)
40 to 49	25.0	2.73 (2.12-3.51)	1.96 (1.51-2.56)
50 to 59	24.1	3.13 (2.44-4.02)	1.94 (1.48-2.55)
60 to 69	30.1	2.85 (2.17-3.75)	1.63 (1.20-2.20)
70 or more	14.7	1.80 (1.31-2.48)	1.00 (0.71-1.43)
Macro-region			
North	9.8	1.00	1.00
Northeast	13.7	1.44 (1.14-1.82)	1.31 (1.04-1.67)
Center-west	14.1	1.63 (1.24-2.14)	1.34 (1.01-1.76)
Southeast	24.1	2.14 (1.70-2.69)	1.75 (1.38-2.22)
South	32.5	2.97 (2.34-3.76)	2.16 (1.68-2.79)
Marital status			
Married or living with a partner	22.3	1.00	1.00
Single or no partner	19.4	1.08 (0.95-1.22)	1.11 (0.97-1.27)
Education			
None or incomplete primary school	22.2	1.00	1.00
Complete primary school or incomplete high school	18.1	0.90 (0.74-1.09)	1.02 (0.83-1.25)
Complete high school or incomplete college/university	18.0	0.90 (0.77-1.05)	1.09 (0.92-1.30)
Complete college/university	29.7	1.46 (1.20-1.78)	1.37 (1.11-1.70)
Multimorbidity			
None	11.0	1.00	1.00
One	18.9	1.69 (1.35-2.10)	1.52 (1.22-1.90)
Two	23.8	2.68 (2.16-3.32)	2.28 (1.82-2.86)
Three or more	31.9	3.45 (2.82-4.22)	2.83 (2.27-3.53)

^a^ *PR* Prevalence Ratio

^b^ Adjusted for all variables shown in Table 2

## Discussion

This is the first study to present nationally representative estimates of access to treatment for depression, and predictors of access among Brazilians with depression. Approximately 80 % of individuals with clinically relevant depressive symptoms have not received any treatment, with the Northern region having the highest proportion of untreated individuals and the Southern region having the lowest proportion. For those receiving treatment, pharmacotherapy only was most common, especially among individuals from the Southern region. With respect to acquisition of medicines, most people with depression had access to free drugs in the public health system (47.4 %), and this proportion was markedly higher in the South (54.6 %) and lower in the North (34.8 %). However, a considerable portion of individuals had to pay for their medicines (41.8 %). It is noteworthy that in three regions (North, Midwest and Southeast), the proportion of individuals who had to buy medicines for depression was higher than that of those who obtained their medications for free.

Our findings are in accordance with previous studies. Wittayanukorn et al. [2], using NHANES data, estimated the prevalence of depressive symptoms and type of treatment across PHQ-9 symptom severity in the U.S. population. They found that approximately 70 % of individuals with any depressive symptoms and over half of those with moderate to severe depression did not receive any treatment. Other studies have also called attention to the low and inadequate access to depression treatment [13–17]. Kessler et al. [16], in a household survey of psychiatric disorders among adult residents in the U.S. found that more than half (57 %) of respondents with 12-month major depressive disorder (MDD) received any treatment in the 12 months before the interview, and the treatment received was considered adequate for only 20.9 % of these individuals. In a systematic assessment of patients with major depression presenting for treatment, Kocsis et al. [17] showed that only 33 % of the 801 registered participants had ever had a prior adequate course of antidepressant medication. Young et al. [13], using data from a cross-sectional telephone survey that included 1636 adults with probable depressive or anxiety disorder in the USA, found that 83 % of them saw a health care provider, but only 30 % received appropriate treatment.

In the present study, women, white individuals, those with college/university degrees, and those presenting with multi-morbidities were more likely to have access to treatment for depression. These findings reveal important inequities in access to treatment among individuals with depression in Brazil and are broadly consistent with those of other studies [2, 17]. The study conducted by Wittayanukorn et al. [2] found similar treatment disparities in the U.S. population, showing that among patients with moderate to severe depression, being female, white, and having comorbidities, increased the likelihood of receiving treatment. Kocsis et al. [17] also showed that patients who were white or had comorbidities were significantly more likely to have received prior adequate antidepressant treatment. Young et al. [13] found that appropriate treatment was less likely for men and those who were black and less educated compared to their female, white and more educated counterparts. A population-based, long-term prospective cohort study conducted by Hengartner et al. [18] including 4,547 adults from Zurich showed that the weighted treatment prevalence for any depressive disorder was 23.4 % (15.7 % for MDE, 4.3 % for minor depressive disorders and 3.4 % for non-diagnosed subjects), and that women with MDE had a treatment prevalence three times larger than that of men (23.8 vs 7.4 %).

In the present study, despite the higher prevalence of depression among non-white skinned individuals, we found that being white increased the likelihood of receiving any treatment for depression. Evidence of an association between skin color and access to depression treatment has also been reported in other studies that evaluated specifically racial/ethnic disparities. For instance, Simpson et al. [19], in a systematic review of the literature to evaluate racial disparities in the diagnosis and treatment of depression in the U.S., found lower rates of treatment for African Americans and Hispanics when compared to Caucasians. Also, Alegría et al. [20], using nationally representative data in a study conducted in the U.S. population, found that among those reporting a depressive disorder in the past year, Latinos, Asians, and African Americans had less access to any mental health treatment when compared to non-Latino whites.

Education has been considered an important factor associated with better access to adequate treatment for depression. Witt et al. [21] using data from the 1996–2005 Medical Expenditure Panel Survey to evaluate access to adequate treatment among mothers with depression, found that those with less education were less likely to receive treatment. The HealthCare for Communities (HCC) study also showed that among individuals with a depressive or anxiety disorder, receipt of appropriate care was significantly more likely among individuals with more years of education [13].

Living in the Northern region was associated with lower likelihood of receiving treatment for depression, independently of individual characteristics. This probably reflects Brazilian regional inequities in the distribution of health services and specialized health care professionals. In fact, a recent survey indicated that in the Southeast region, where 43.8 % of the Brazilian population lives, there were 4,104 psychiatrists, corresponding to one psychiatrist for every 15,000 people. In contrast, the North region, where 7.4 % of the population lives, had only 134 psychiatrists, corresponding to one psychiatrist for more than 80,000 people [22].

### Limitations

Our study has some limitations. Presence of depression was assessed with the PHQ-9, and pooled estimates of sensitivity and specificity for the cut-off 10 are 0.77 and 0.85, respectively, implying that some degree of random misclassification occurred. This may have diluted observed associations. Access to treatment was based on self-report, and individuals with higher socioeconomic conditions may have better awareness of medical diagnoses and treatments they receive. We were not able to control for some relevant characteristics, such as income, therefore some of the observed associations may be affected by uncontrolled confounding.

### Implications

Several strategies must be implemented in order to overcome barriers, improve access to treatment for depression, and reduce health care inequities in Brazil. Such strategies involve actions to overcome attitudinal barriers, which are related to personal beliefs and social stigma, and structural barriers, related to the way health care is delivered and accessible to the population [23]. In Brazil, as in many low- and middle-income countries, there is an urgent need for dissemination of information about depression and its treatments, in order to increase awareness and reduce social stigma, which is still very high [24]. Regarding structural barriers, integrating mental health into primary care is essential, and must involve several strategies. In Brazil, primary care covers most of the population [25]. However, primary care staff are still largely ill-prepared to deal with mental health problems. Screening for depressive symptoms is important but not effective by itself. Task-shifting strategies that allow less specialized primary care staff to deliver effective treatment need to be developed and tested. Public policies must target vulnerable groups, such as non-white individuals, in order to reduce inequities related to ethnicity/skin color. Special attention must be paid to men’s health programs, since men are less likely to seek help and health care staff are not well prepared to identify they health care needs, including the need for mental health care [26].

## Conclusion

In Brazil, access to treatment is very low, and those most vulnerable are less likely to receive care for depression. Tackling this important health inequity requires a series of actions and public policies aimed at overcoming barriers to access to mental health care.
